# Cell wall biochemical alterations during *Agrobacterium*‐mediated expression of haemagglutinin‐based influenza virus‐like vaccine particles in tobacco

**DOI:** 10.1111/pbi.12607

**Published:** 2017-01-05

**Authors:** François Le Mauff, Corinne Loutelier‐Bourhis, Muriel Bardor, Caroline Berard, Alain Doucet, Marc‐André D'Aoust, Louis‐Philippe Vezina, Azeddine Driouich, Manon M.‐J. Couture, Patrice Lerouge

**Affiliations:** ^1^ Laboratoire Glyco‐MEV EA 4358UNIROUENNormandie UnivRouenFrance; ^2^ Medicago Inc.QuébecQCCanada; ^3^ Laboratoire COBRAUNIROUENCNRS UMR 6014Normandie UnivRouenFrance; ^4^ UNIROUENLITIS EA 4108Normandie UnivRouenFrance; ^5^ Present address: Departments of Medicine, Microbiology and ImmunologyMcGill universityMontrealQCCanada; ^6^ Present address: Infectious Diseases in Global Health ProgramResearch Institute of the McGill University Health CentreMcGill UniversityMontrealQCCanada; ^7^ Present address: Groupe TH Inc. 1327, avenue Maguire, suite 100QuébecQCG1T 1Z2Canada

**Keywords:** cell wall, virus‐like particle, plant, Agrobacterium, tobacco, haemagglutinin

## Abstract

Influenza virus‐like particles (VLPs) have been shown to induce a safe and potent immune response through both humoral and cellular responses. They represent promising novel influenza vaccines. Plant‐based biotechnology allows for the large‐scale production of VLPs of biopharmaceutical interest using different model organisms, including *Nicotiana benthamiana* plants. Through this platform, influenza VLPs bud from the plasma membrane and accumulate between the membrane and the plant cell wall. To design and optimize efficient production processes, a better understanding of the plant cell wall composition of infiltrated tobacco leaves is a major interest for the plant biotechnology industry. In this study, we have investigated the alteration of the biochemical composition of the cell walls of *N. benthamiana* leaves subjected to abiotic and biotic stresses induced by the *Agrobacterium*‐mediated transient transformation and the resulting high expression levels of influenza VLPs. Results show that abiotic stress due to vacuum infiltration without *Agrobacterium* did not induce any detectable modification of the leaf cell wall when compared to non infiltrated leaves. In contrast, various chemical changes of the leaf cell wall were observed post‐*Agrobacterium* infiltration. Indeed, *Agrobacterium* infection induced deposition of callose and lignin, modified the pectin methylesterification and increased both arabinosylation of RG‐I side chains and the expression of arabinogalactan proteins. Moreover, these modifications were slightly greater in plants expressing haemagglutinin‐based VLP than in plants infiltrated with the *Agrobacterium* strain containing only the p19 suppressor of silencing.

## Introduction

The market share of vaccines is rapidly increasing with a growing interest towards virus‐like particles (VLPs) products. VLPs are produced by recombinant expression in heterologous systems of viral structural proteins. They mimic viruses with respect to their morphology and size as well as their immunogenic potential, but do not contain viral DNA or RNA constituting an important safety advantage over traditional vaccines (Grgacic and Anderson, 2006; Le Mauff *et al*., 2014; Lua *et al*., 2014). The last two decades witnessed the rise of plant biotechnology and demonstrated the potential of plant‐derived biopharmaceutical production platforms. Stable or transient expression of proteins in plants allows for the production of well‐folded and functional biopharmaceuticals, including VLPs, in a safe and scalable manner (Kushnir *et al*., 2012). Medicago Inc. has developed a plant‐based VLP manufacturing platform utilizing the transient *Agrobacterium*‐mediated expression of haemagglutinin in *Nicotiana benthamiana* capable of efficiently producing influenza VLPs (D'Aoust *et al*., 2008, 2010). Through this platform, influenza VLP expression and purification technologies were brought to large‐scale manufacturing of GMP‐grade pharmaceutical products. As with the influenza virus, plant‐made influenza VLPs acquire a lipid envelop during the particle budding process, but the latter only contain the haemagglutinin immunogenic determinants of the influenza virus (Vézina *et al*., 2011). Transmission electron microscopy imaging of *N. benthamiana* leaves expressing VLPs indicated that the candidate vaccines accumulate out of the plant cell, between the plasma membrane and the cell wall (D'Aoust *et al*., 2008; Vézina *et al*., 2011). As agro‐infiltration‐based production of VLPs requires *Agrobacterium tumefaciens* as a vehicle for gene shuttling to the host cell nucleus, studying the impact of *Agrobacterium* infiltration on plant tissues composition is of primary importance. Although the plant–*Agrobacterium* interaction is well understood, limited knowledge exists on the cell wall modifications occurring during transient gene expression (Pitzschke and Hirt, 2010; Pruss *et al*., 2008). Therefore, understanding the cell wall biochemical modification in tobacco leaves during the production process of VLP vaccines is of great interest.

Plant cell wall is composed of three main polymers: cellulose, hemicellulose and pectins. Cellulose is a polymer of β(1,4)‐glucose, which constitutes the main network of the cell wall ensuring anisotropic growth of cells (Baskin and Jensen, 2013; Brown, 2004). Hemicellulose encompasses polymers of β(1,4)‐linked monosaccharides substituted by various side chains. Among them, xyloglucans are the most abundant hemicellulose in primary cell walls of eudicots. They consist of a cellulose backbone substituted by α(1,6)‐linked xylose residues and additional arabinose, galactose or fucose units (Pena *et al*., 2008). Acetylesterification occurs either on carbon 6 of the glucose backbone, or on the galactose or arabinose residues of the side chains (Gille *et al*., 2011). The xylans and mannans hemicelluloses are less abundant in eudicots and do not exceed 5% of total hemicelluloses (Scheller and Ulvskov, 2010). Pectins are acidic polymers of α(1,4)‐linked homogalacturonan (HG) and rhamnogalacturonans (RG). HG presents different levels of methyl‐ and acetylesterification which varies according to developmental stage of the plant (Wolf *et al*., 2009). RG is further subdivided into two classes: RG‐I which consists of arabinan and galactan chains linked to a backbone of [→2)‐α‐L‐Rha*p*‐(1→4)‐α‐D‐Gal*p*A‐(1→] repeating disaccharides (Mohnen, 2008), and RG‐II which possesses a homogalacturonan backbone substituted with four complex and structurally conserved oligosaccharide side chains (O'Neill, 2001). Finally, in addition to these polysaccharides, the cell wall contains hydroxyproline‐rich glycoproteins (HRGP) (Nguema‐Ona *et al*., 2013) such as arabinogalactan proteins (AGPs) and structural proteins.

Infection of plants by pathogens induces a large set of plant defence responses including deposition of lignin and callose, as well as cross‐linking between cell wall polysaccharides and glycoproteins (Malinovsky *et al*., 2014). These chemical modifications reinforce the walls of the cells surrounding the infection site, creating a barrier that limits the spread of the pathogen (Zhao and Dixon, 2014). To date, modifications of cell wall polymers under biotic stress conditions are well described (Malinovsky *et al*., 2014): for example, the relationship between defence signal pathways, such as the jasmonic acid pathway, and modification of cellulose synthesis and lignification have been clearly demonstrated (Caño‐Delgado *et al*., 2003). Moreover, xylan networks are known to be reinforced during infection through the cross‐linking of ferulate esters with lignins (Malinovsky *et al*., 2014). Pectins also play a key role as sentinels of the cell wall integrity: in the context of pathogen–plant interactions, pathogen and plant pectin‐degrading enzymes release partially methyl‐esterified oligogalacturonide fragments that act as signals of tissue infection by pathogens (Bethke *et al*., 2014).

In this study, we present the biochemical alterations of the cell wall of *N. benthamiana* plants arising from abiotic stress resulting from the vacuum infiltration as well as from biotic stresses caused by infiltration of *Agrobacterium* with or without the transient expression of influenza VLPs.

## Results

### Experimental design

**Figure 1:**
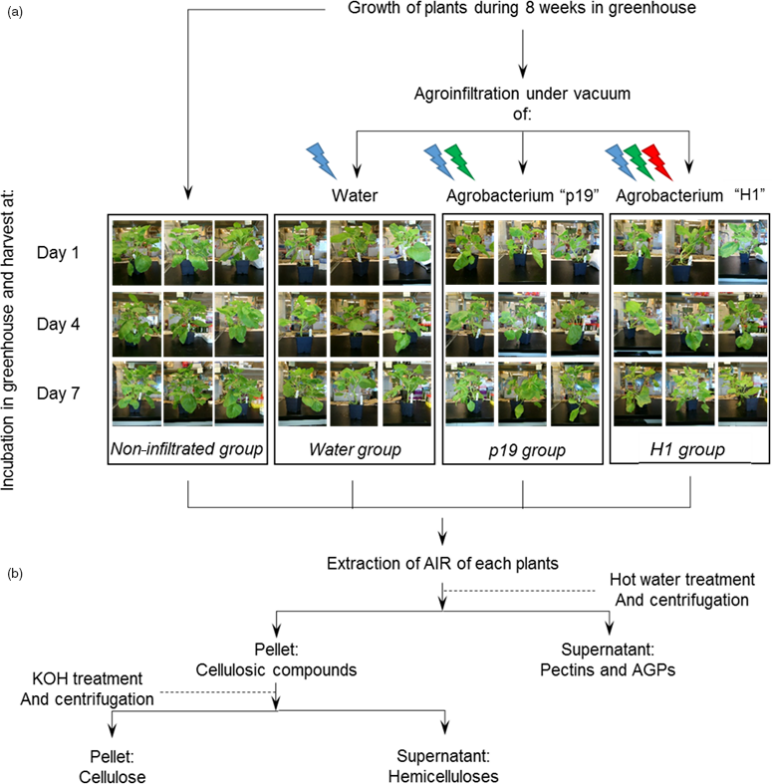
Experimental design of plants submitted to different biotic and abiotic treatments (a) and preparation of the cell wall constituents (b). Lightened icons represent the different stresses applied to each groups: abiotic stress from infiltration is in blue, biotic stress of bacterial infection is represented in green, and biotic stress arising from VLP production and accumulation is in red.

Four *N. benthamiana* plant groups were submitted to different abiotic and biotic stresses in order to analyse their respective impact on plant cell wall constituents (Figure 1a). Sugar composition and linkage analyses of cell walls prepared from agro‐infiltrated and noninfiltrated *N. benthamiana* plants were carried out to investigate the influence of *Agrobacterium*‐mediated influenza VLP production on the cell wall composition (Figure 1b). Cell walls prepared from non infiltrated (Ni) plants subjected to the same conditions as the three other groups were analysed and considered as a non infiltrated control group, while cell walls of plants infiltrated with water only (Water) were studied to investigate the impact of the abiotic stress due to the vacuum treatment used to infiltrate the *Agrobacterium* inside plant tissues. A third group of plants was agro‐infiltrated with an *Agrobacterium* strain carrying a binary plasmid, designed for the expression of the p19 suppressor of gene silencing (labelled as ‘p19’ plants), to assess biotic stress due to *Agrobacterium* infection in the absence of VLP expression. Finally, a last plant group was infiltrated with an *Agrobacterium* strain capable of transferring both p19 and the *Influenza* H1 haemagglutinin genes to the plant cells allowing for transient expression of the haemagglutinin and budding of VLP (annotated as ‘H1’) (Figure 1a). For each group, leaves from three plants were harvested on days 1, 4 and 7 post‐infiltration and the different cell wall components were sequentially extracted as follow: alcohol insoluble residues (AIR) were first prepared and mainly consisted of cell wall polysaccharides and AGPs. Then, water‐soluble constituents (pectins and AGPs) and hemicellulose fractions were extracted from the AIR fraction of each plant. Finally, residual insoluble material was considered as being insoluble cellulose (Figure 1b) (Coimbra *et al*., 1985). Confirmation of expression of the H1 VLP was carried out at day 7 on three biological replicates (Figure S1).

### Overall sugar composition of crude cell walls extracts (AIR)

**Figure 2:**
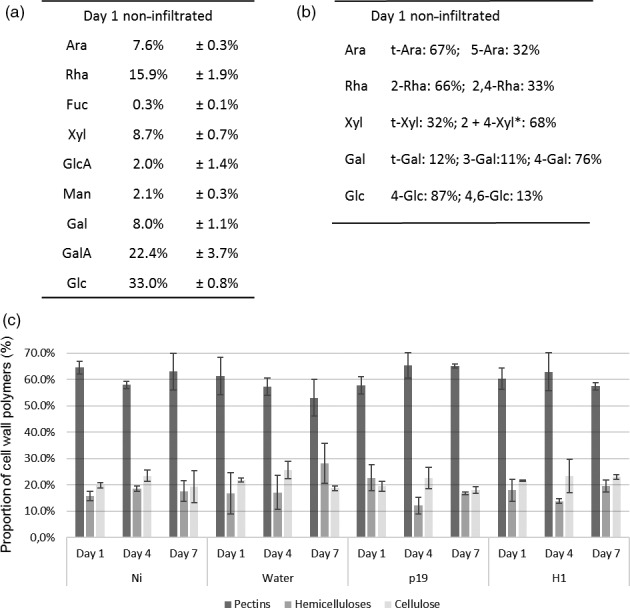
Analysis of AIR material. Sugar composition (a) and linkages (b) analysis of main neutral monosaccharides of AIR extracted from control leaves at day 1. *Co‐eluting partially O‐methylated samples. Relative percentages of cell wall polysaccharide fractions from all conditions at days 1, 4 and 7 (c). Ara: arabinose, Rha: rhamnose, Fuc: fucose, Xyl: xylose, GlcA: glucuronic acid, Man: mannose, GalA: galacturonic acid, Gal: galactose, Glc: glucose, t‐: terminal.

Monosaccharide composition of AIR isolated from cell walls of non infiltrated *N. benthamiana* leaves (Ni) was in agreement with previous data (Figure 2a) (Nguema‐Ona *et al*., 2012) showing that glucose, galacturonic acid and rhamnose are the most abundant monosaccharides. Similar monosaccharide compositions were measured in AIR isolated from water, p19‐ and H1‐infiltrated leaves collected from day 1 to day 7 (Table S1). Sugar linkage composition of neutral monosaccharides, identified by gas chromatography coupled to an electron ionization mass spectrometer (GC‐EIMS) after permethylation of the sample, was first determined in the Ni group. In the cell wall AIR fraction of these plants, the most abundant partially methylated alditol acetate derivatives were assigned to 4‐linked glucose, 4,6‐linked glucose, 2‐ and/or 4‐linked xylose and terminal xylose derived from cellulose and xyloglucans (Figure 2b). Detection of 2‐linked rhamnose and 2,4‐linked rhamnose residues in a 2/1 ratio indicated that a third of the [→2)‐α‐L‐Rha*p*‐(1→4)‐α‐D‐Gal*p*A‐(1→] repeating units of the RG‐I backbone of cell walls of *N. benthamania* leaves were substituted by side chains. Linkages of galactose and arabinose residues were consistent with the presence of branched 5‐linked arabinans and 4‐linked galactans (Figure 2b).

The three main cell wall fractions, pectins, hemicelluloses and cellulose, isolated from AIR, were quantified for each plant group. The relative abundance of pectin, hemicellulose and cellulose components varied from 53 to 65%, from 12 to 28% and from 18 to 26%, respectively. No statistical significant differences were observed between control (Ni) and water, p19‐ and H1‐infiltrated plants collected at days 1, 4 and 7 (Figure 2c).

Together, monosaccharide composition and sugar linkages analyses indicated that no major overall change in the cell wall polysaccharide composition is occurring in infiltrated plants expressing H1 VLP over the seven‐day incubation period, when compared to the control groups subjected to different stresses (Ni, water and p19). The plant cell wall fractions were further analysed to investigate whether subtle modifications were induced by the different plant treatments.

### Analysis of pectins and AGPs

**Figure 3:**
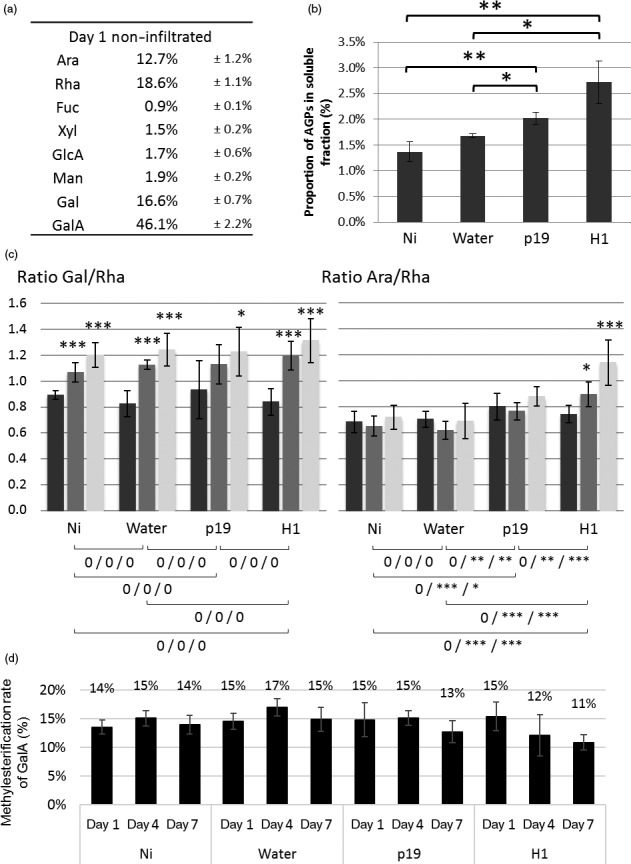
Analysis of water‐soluble material. Monosaccharide composition of water‐soluble fraction of control plant at day 1 (a). Quantification of AGPs present in the water‐soluble fraction at day 7 (b). *: *P*‐value below 0.05, **: *P*‐value below 0.01 and ***: *P*‐value below 0.001. Gal/Rha (left panel) and Ara/Rha ratio (right panel). Day 1 in black, day 4 in grey and day 7 in white. Symbols below charts indicate statistical analysis between the different conditions. First symbol corresponds to analysis performed on day 1, second one on day 4 and last one on day 7 (c). 0: no difference, *: *P*‐value below 0.05, **: *P*‐value below 0.01 and ***: *P*‐value below 0.001. Degrees of methylesterification of pectins (D).

Monosaccharide composition of polysaccharides extracted in hot water was further investigated to determine whether the different infiltration treatments could induce subtle structural modifications which would not have been revealed in the analysis of overall AIR fractions. This hot‐water‐soluble fraction mainly contained pectins and AGPs of the cell wall. Figure 3a presents the monosaccharide composition obtained for the Ni plant group at day 1. Galacturonic acid (GalA) was the main monosaccharide present in this fraction, followed by galactose (Gal), arabinose (Ara) and rhamnose (Rha). This indicated that pectins were mainly constituted of RG‐I and homogalacturonans. Other pectic polysaccharides, such as RG‐II and xylogalacturonans, represented only a small fraction of total pectins extracted and were not further analysed.

Quantification of AGP was carried out by rocket gel electrophoresis, which showed that AGPs represented only 1.5 to 3% (w/w) of the water‐soluble fractions (Figure 3b). Furthermore, as shown in Figure 3b, the proportion of AGPs was found to increase by two fold for H1‐infiltrated leaves when compared to Ni and water groups. The contribution of the AGP to the monosaccharide composition was therefore revealed to be quite low. In consequence, monosaccharides detected in the hot‐water‐soluble fraction mainly arise from pectins.

Sugar composition of the pectins extracted in the hot‐water‐soluble fractions showed slight variations between tobacco leaf groups and over time. To better understand and illustrate these observations, three different ratios were calculated from the relative amount of the main sugars components: the GalA/Rha ratio reflects the proportion between RG‐I and HG as these two monosaccharides are unique monomers of these respective pectin backbones, whereas ramification sizes of RG‐I can be represented by the ratios of Ara or Gal (monosaccharides present in the side chains) relative to Rha residues (constituent of the RG‐I backbone). GalA/Rha ratio indicated that RG‐I and HG were found in similar amounts in pectin extracts of all the plant groups with ca 58% of RG‐I and 42% of HG. In contrast, Gal/Rha and Ara/Rha ratios changed over time or among plant groups. Indeed, the Gal/Rha ratio increased from day 1 to day 7 in all plant groups and likely revealed the increase of galactan ramifications of the RG‐I during the growth of plants over the seven‐day incubation period (Figure 3c, left panel). In contrast, the increase in Ara/Rha ratio was observed only in agro‐infiltrated leaves (p19 and H1) and not in the Ni leaves nor in the leaves infiltrated with water (Figure 3c, right panel). Moreover, this increase was greater in leaves expressing haemagglutinin‐based VLP (H1). It was then concluded that infiltration with *Agrobacterium* strains induces an increase of arabinosylation of cell wall RG‐I constituent, with an even higher increase when the haemagglutinin‐based VLP is expressed.

Pectins are usually methyl‐ and/or acetyl‐esterified. Degrees of acetylesterification and methylesterification were therefore measured in the different pectin fractions. No modification of the level of acetylation was detected suggesting that acetylesterification of pectins was not affected by any of the treatment performed (data not shown). As shown in Figure 3d, overall degree of methylesterification of the pectin was ca 15% and remained constant over the seven‐day incubation period in Ni, water and p19 groups. Interestingly, this level of methylesterification slightly decreased from 15% to 11% over the same incubation period for the H1 group.

### Analysis of hemicelluloses

**Figure 4:**
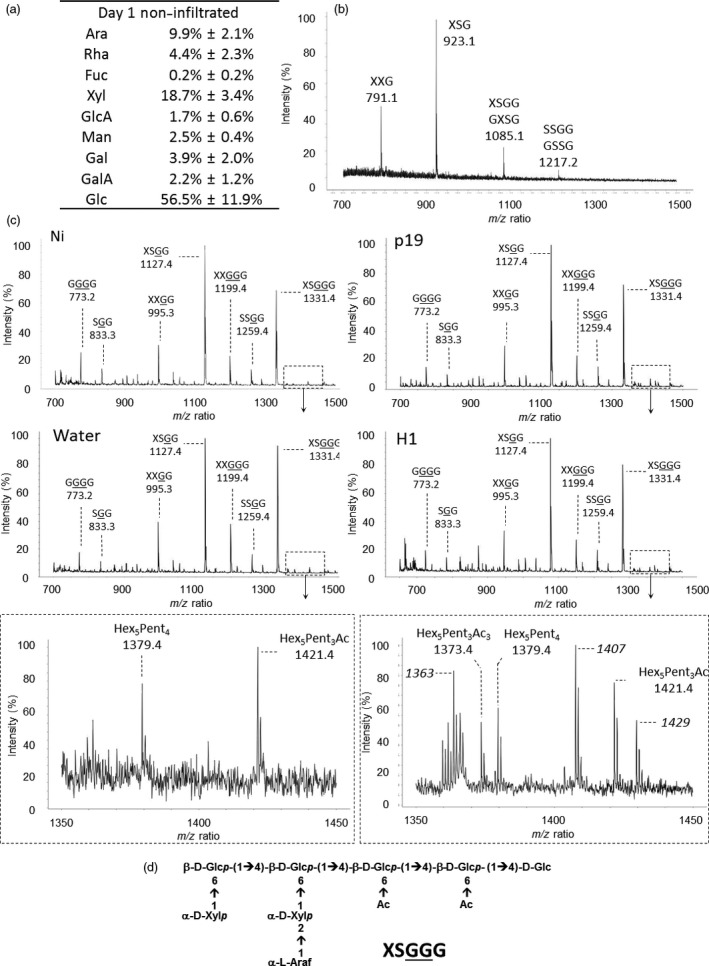
Analysis of hemicellulose material. Sugar composition determined by GC‐FID (a) and xyloglucan fingerprint of the hemicellulose fraction isolated from noninfiltrated leaves by MALDI‐TOF MS (b). Xyloglucan fingerprints of AIR isolated from Ni, water, p19 and H1 (c). Structure of the XSGGG fragment (d). Nomenclature of xyloglucan fragments according to Fry *et al*. (1993). G: Glc residue carrying an acetate group.

Monosaccharide composition of hemicellulose fractions from AIR extracted from the four plant groups over time was investigated. In addition, fragments of hemicelluloses released by digestion with specific endoglycanases were analysed by matrix‐assisted laser desorption ionization–time‐of‐flight mass spectrometry (MALDI‐TOF MS). This approach produces enzyme‐specific fingerprints which allow for a fast and informative monitoring of hemicellulose structural variations (Lerouxel *et al*., 2002). Sugar composition of hemicellulose indicated that the main monosaccharides found were glucose (Glc), xylose (Xyl) and arabinose (Ara), in a 5:2:1 ratio (Figure 4a) as expected according to literature (York *et al*., 1996). Moreover, this ratio remained constant in the different groups studied as well as over time.

The presence of xylans was investigated by digesting hemicellulose extracts with an endoxylanase and analysing the resulting xylan fragments by MALDI‐TOF MS. No fragment corresponding to the xylan fingerprint was detected indicating that these hemicelluloses are in trace amounts in *N. benthamiana* cell wall (data not shown). In contrast, as illustrated in Figure 4b, digestion of the hemicellulose fractions with an endoglucanase, that cleaves xyloglucan after non substituted glucose residues, released three main fragments for which masses were assigned to XXG, XSG and XSGG/GXSG, in agreement with data reported in the literature (Chevalier *et al*., 2010; Sims *et al*., 1996; York *et al*., 1996). These fragments were named according to the nomenclature suggested by Fry *et al*. (1993) where G refers to glucose units of the backbone, X and S refer to α‐D‐Xyl*p*‐(1→6) and to α‐L‐Ara*f*‐(1→2)‐α‐D‐Xylp‐(1→6) xyloglucan side chains, respectively (Figure 4d). The same enzyme fingerprinting analyses were performed on hemicellulose fractions isolated from leaves of plants harvested at days 1, 4 and 7. No detectable modification of monosaccharide composition and MALDI‐TOF MS profiles was observed across the different plant groups (Table S2A).

To investigate the presence of alkali labile substitutions on xyloglucan, endoglucanase digestion was performed on AIR fractions. In these conditions, endoglucanase releases fragments from native xyloglucan carrying alkali labile groups that are otherwise lost during hemicellulose extraction with 4 M KOH. MALDI‐TOF MS analysis of the digests showed xyloglucan fragments carrying one or two acetate groups (+ 42 Da) and larger oligoglycosyl backbones (Figure 4c, first panel, Table S2B) (Chevalier *et al*., 2010; Sims *et al*., 1996). The presence of these fragments indicated that acetylation occurs on glucose residues that protect the backbone from exhaustive endoglucanase digestion. Based on these data, it was concluded that xyloglucan from *N. benthamania* cell wall leaves mainly consists of XXGGG repeating units that are substituted on xylose side chains by arabinose residues and acetylated on the backbone glucose residues (Figure 4d). The structure of major fragments was confirmed by their fragmentation patterns obtained from MALDI‐TOF MS/MS (Figure S2 in case of *m/z* 1331.4). Endoglucanase xyloglucan fingerprinting was carried out on AIR isolated from infiltrated leaves collected at days 1, 4 and 7 (Figure 4c). In the agro‐infiltrated leaves (p19 and H1), minor ions were specifically and reproducibly detected in the *m/z* 1350‐1450 range (Figure 4c). These ions were neither detected in control leaves nor in leaves infiltrated with water, indicating that these additional xyloglucan fragments resulted from alkali labile modifications of xyloglucan in response to *Agrobacterium* infiltration. These ions, that are the unique modifications observed in xyloglucan fingerprints, did not significantly modify the xyloglucan overall structure and remain unidentified (Tables S3B and S2C). Nevertheless, a significant increase of monolignols was observed in the xyloglucan‐enriched fraction of H1 group at day 7 compared to Ni group at the same day (data not shown).

### Callose and lignin deposition

**Figure 5:**
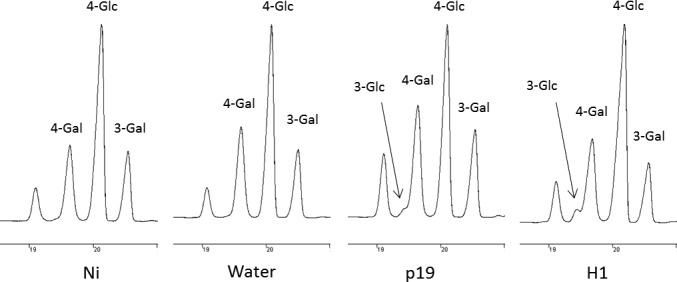
Callose detection. Gas chromatograms of the monosubstituted hexose regions (18.5–21 min) obtained after permethylation of AIR isolated from leaves collected at day 7 from control plants (Ni) and plants infiltrated with water or *Agrobacterium* strains (p19 and H1); 3‐linked Glc results from callose deposition; 4‐linked Glc corresponds to cellulose and 3‐linked and 4‐linked Gal arises from galactans.

Callose deposition was investigated by staining the tissue with aniline blue and observing the presence of callose under confocal microscope. As expected, this revealed that callose is synthesized in response to the agroinfiltration stress (not shown). To quantify the extent of callose deposition in infiltrated plants, 3‐linked Glc was measured by GC‐MS in leaves collected from day 7 plants. Permethylation of polysaccharides of AIR fractions allows the discrimination between 4‐linked Glc derived from cellulose and 3‐linked Glc found in callose only. Figure 5 shows the gas chromatography profiles of the monosubstituted hexoses obtained from day 7 leaves of Ni and water, or p19‐ or H1‐infiltrated leaves. The GC‐FID analyses of PMAA demonstrated the presence of 3‐linked Glc and therefore reflect that callose is specifically synthesized in agro‐infiltrated leaves (p19 and H1). Relative quantification of 3‐linked Glc showed that callose represented about 3% (w/w) of the cell wall polysaccharides in infected tissues of H1 VLP‐expressing plants.

**Figure 6:**
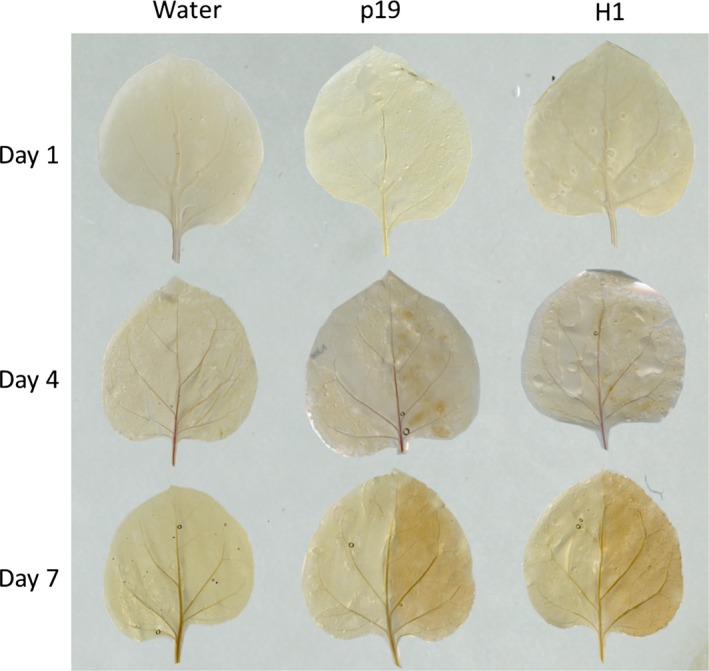
Lignin detection at days 1, 4 and 7 by staining with phloroglucinol. For each plant group, the right side of the leaves was infiltrated, whereas the left side was not.

Lignin deposition was monitored by phloroglucinol staining of leaves. Infiltrations were carried out with a syringe on one‐half of the leaf; the other half remained untreated for comparison (Figure 6). Lignin deposition was detected as brown spots in agro‐infiltrated leaves (p19 and H1) from day 4. At day 7, a more contrasted staining of all the infected tissues was observed relative to their respective control non treated half leaf. In contrast, leaves infiltrated with water were comparable to control leaves demonstrating the specificity of the signal obtained.

## Discussion

Plant‐based biotechnology allows for the large‐scale production of Influenza virus‐like particles in *N. benthamiana* leaves through the *Agrobacterium*‐mediated transient transformation and the expression of haemagglutinin surface protein (D'Aoust *et al*., 2008, 2010). To investigate to what extent abiotic stress due to vacuum infiltration and/or biotic stress due to agro‐infiltration induce cell wall modifications, chemical composition of cell walls from *N. benthamania* leaves was analysed, in plants infiltrated under vacuum with different inoculum solutions (water, p19, H1) over a seven‐day postinfiltration period and results were compared to those of noninfiltrated plants. This study demonstrated that stresses induced by the infiltration process brought to large scale for transient expression of considerable amounts of biomass were not causing significant cell wall modification at the macromolecular level. All plant groups analysed showed a predominant portion of pectins in their cell wall and cellulosic compounds split equally between arabinoxyloglucans as the main hemicellulose, and cellulose, as it was expected for the *N. benthamiana* plants (Nguema‐Ona *et al*., 2012; York *et al*., 1996). Observations of significant modifications were only possible after fractionation of the different polymers constituting the cell wall.

Looking at structural components of pectins, two main modifications were observed. The first one was a decrease in the methylesterification rate of the homogalacturonans. This process is already described in the literature and relies on the fact that during plant–pathogen interactions, homogalacturonan oligosaccharides are involved in the elicitation of the plant defence responses. Through the action of pectin‐degrading enzymes, weakly methyl‐esterified oligogalacturonides resulting from the degradation of homogalacturonans are recognized by wall‐associated kinases and induce defence gene expression (Ferrari *et al*., 2013; Osorio *et al*., 2008). Moreover, de‐esterification of galacturonic acid residues by pectin methylesterases is known to promote cell wall rigidification resulting in the cross‐linking of the acidic pectins with calcium ions, affecting the porosity of the primary cell wall and thus forming a stronger barrier to cross for pathogens (Peaucelle *et al*., 2012). The second modification observed on this polymer was on the ramifications of the rhamnogalacturonan. Analysis of the different ratio Ara/Rha and Gal/Rha exposed two different pectin responses. Galactosylation was shown to increase in relation with the growth of the plant as all groups showed a significant increase after the 4‐day and 7‐day incubation duration. Arabinosylation level of the rhamnogalacturonan side chains was increased in *Agrobacterium*‐infiltrated leaves (p19 and H1 plants) by comparison with control plants. Such a cell wall modification in response to an *Agrobacterium* infection was not reported before. Elongation of RG‐I arabinan chains during infection could reinforce the primary cell wall in response to infection as these side chains are known to interact with cellulose or xyloglucan (Wang *et al*., 2012; Zykwinska *et al*., 2005). Moreover, synthesis of arabinans may impact the flexibility of cell wall. Indeed, it was previously reported that, in an abiotic stress such as dehydration, arabinans are involved in the plant survival modulating the flexibility of the cell wall (Moore *et al*., 2008, 2013).

The level of AGP was also increased at day 7 in agro‐infiltrated leaves in comparison with non infiltrated plants. Although the involvement of AGPs in plant–pathogen interaction has already been mentioned in the literature (Nguema‐Ona *et al*., 2013; Seifert and Roberts, 2007), their precise role in the metabolic pathway of the plant defence against pathogens remains unclear. It was demonstrated that they can link pectins and hemicelluloses together and therefore may contribute to the cell wall rigidification, thus limiting the spread of the pathogens in the leaf tissue (Popper and Fry, 2007; Tan *et al*., 2013). Another demonstration performed by Cannesan *et al*., 2012 also established the involvement of the AGPs in plant defence reaction, highlighting the role of these molecules in the control of early infection of pea roots. In the industrial context of plant infection by *Agrobacterium*, the role of AGPs in the defence mechanisms remains unknown, but our results exposed a potential role of these molecules in the leaf tissue as their population doubled over the seven‐day infection.

Hemicelluloses in *N. benthamiana* cell wall are mainly composed of arabinoxyloglucans consisting of XXGGG repeating units that are substituted on xylose side chains by arabinose residues and acetylated on glucose residues of the backbone, in agreement with data reported for hemicelluloses from Solanaceae plants (Vincken *et al*., 1997; York *et al*., 1996). Enzymatic hydrolysis of AIR with endo‐β‐1.4‐glucanase and MALDI‐MS enzyme fingerprinting enabled the detection of numerous xyloglucan fragments carrying acetate groups. Comparison of MS profiles between the different plants groups did not show significant differences in the content of major fragments. Nevertheless, *Agrobacterium* infiltration induced the accumulation of three low abundant xyloglucan fragments that were not detected in control or water‐infiltrated leaves and which structures remain unidentified. As these fragments were no longer observed after alkali treatments and considering that higher amounts of monolignols were detected in xyloglucan fractions, the hypothesis is that these stress‐induced fragments could arise from the esterification of xyloglucan with monolignols. Such esterification has been reported for a dimer Xyl‐α‐1‐6‐Glc of arabinoxyloglucans by Ishii *et al*., 1990. More experiments will have to be carried out to unravel the structures of these minor xyloglucan motifs and determine their function in plant defence against pathogens.

Callose and lignins polymers are known to be deposited in the cell wall during infections. These modifications are known to reinforce the walls of cells surrounding the infection site to limit the spread of the pathogen (Zhao and Dixon, 2014). Callose accounted for up to 3% (w/w) of the cell wall polysaccharides in p19‐ and H1‐infiltrated leaf groups. In addition, phloroglucinol staining revealed lignin deposition between the day 4 and the day 7 in leaves submitted to *Agrobacterium* infiltration treatments only.

Comparison of cell walls of leaves collected from p19 and H1 plants indicated that the expression of H1‐VLP vaccines induced greater cell wall modifications than those observed postinfiltration with *Agrobacterium* containing the inhibitor of gene silencing p19 sequence alone. This is mainly illustrated by data with regard to AGPs, degree of methylesterification and arabinan content. These observations suggested that in addition to defence reactions related to the infection with *Agrobacterium* strains, accumulation of VLPs in leaves may induce additional modifications either due to a physical constraint related to HA accumulation or budding of VLP between the plasma membrane and the cell wall.

Together, these data allow a better understanding of the modifications to plant cell wall composition in infiltrated tobacco leaves in the context of the production of influenza VLPs. As the identified modifications are known to rigidify the cell wall and to alter its degradation by pathogens, it is therefore expected that these modifications have an impact on the mechanical and enzymatic extraction processes. As a further step towards an optimal extraction process, we are currently evaluating the impact of the modifications to cell wall composition on the efficiency of the influenza VLP extraction process. The acquired knowledge on the cell wall structure and its defence‐induced reinforcement will greatly help in designing optimal extraction processes for influenza VLPs and other biopharmaceutical proteins.

## Experimental procedures

### Materials

Eight‐week‐old *N. benthamiana* plants were infiltrated under vacuum either with water or with *Agrobacterium tumefaciens* suspension transformed with the plasmids previously described by D'Aoust *et al*. (2008), encoding for the silencing p19 alone or in combination with a gene sequence encoding the *Influenza* H1 haemagglutinin gene from strain A/California/7/2009. At days 1, 4 and 7 following *Agrobacterium* infiltration, 12 plants were taken out of the green house, three corresponding to non infiltrated plants as negative control group, three plants infiltrated with water and named water, three plants infiltrated with *Agrobacterium* containing the p19 gene sequence named ‘p19’ and finally three plants infiltrated with *Agrobacterium* containing both p19 and H1 haemagglutinin gene sequences named ‘H1’ (Figure 1a).

### Preparation of alcohol insoluble residues

Leaves were harvested and then crushed in 70% ethanol. To remove chlorophyll and pigments, successive incubations were performed in 70% ethanol at 70 °C for 15 min and centrifugation at 4 500 ***g*** for 5 min at 4 °C. Once pigments were completely removed from the samples, the insoluble materials were washed in methanol: chloroform (1: 1) and then in acetone. These successive washing steps led to isolation of insoluble samples named alcohol insoluble residues (AIR) (Figure 1b).

### Sequential extraction of cell wall polysaccharides

Pectins and AGPs were extracted from AIR fraction by incubation in water at 90 °C and then in 0.5% oxalate ammonium at 90 °C. Soluble fractions were separated from the insoluble materials by centrifugation at 5000 ***g***, dialysed against water for oxalate ammonium part and then lyophilized. Hemicelluloses were extracted from the previous insoluble residue with 4 M potassium hydroxide containing 0.1% sodium borohydride. Soluble fractions containing hemicelluloses were neutralized and dialysed against water. Insoluble residues after 4 M KOH treatments correspond to insoluble cellulose.

### Western blot analysis

SDS‐PAGE of protein extracts from H1 plants and transfer onto nitrocellulose membranes were performed as reported in D'Aoust *et al*. (2008). Immunodetection of haemagglutinin was performed using polyclonal antibodies raised against H1 from strain A/Califorina/7/2009 (NIBSC 09/152), and detection with a Rabbit anti‐sheep sera (JIR 313‐035‐045).

### Monosaccharide composition

Two milligrams of polysaccharide fractions was hydrolysed with 2M trifluoroacetic acid (TFA) for 2 h at 110 °C. After freeze‐drying, samples were then converted in methyl glycosides by heating in 1 M methanol‐HCl (Supelco) for 16 h at 80 °C. Samples were dried under a stream of nitrogen, washed twice with methanol and then treated with hexamethyldisilazane (HMDS): trimethylchlorosilane (TMCS): pyridine solution (3: 1: 9, Supelco), for 20 min at 80 °C. The resulting trimethylsilyl methyl glycosides were dried, resuspended in 1 mL of cyclohexane and injected in the 3800 GC system equipped with a CP‐Sil5‐CB capillary column (Agilent Technologies). Elution was performed with the following temperature gradient: 120 °C to 160 °C at a rate of 10 °C/min, 160 °C to 220 °C at a rate of 1.5 °C/min, 220 °C to 280 °C at a rate of 20 °C/min. Quantification of each monosaccharides was carried out using standards and response factors determined for each monosaccharide.

### Sugar linkage analysis

Five milligrams of AIR was permethylated with iodomethane in a suspension of NaOH in dry DMSO, and the resulting partially methylated alditol acetate derivatives were prepared according to protocols described by the glycotechnology core resource of SanDiego (http://glycotech.ucsd.edu/protocols/07_Comp_Analysis_by_Alditol_Rev2.pdf). Monosaccharide derivatives were injected in a gas chromatograph (GC) (Hewlett‐Packard 6890 series) coupled to an Autospec mass spectrometer (MS) (Micromass, Manchester, UK) equipped with an electron ionization (EI) source and the Opus 3.1 data system. Separations were obtained using a Zebron Z5‐MSi (30 m, 0.25 mm id, 0.25 μm film thickness, Phenomenex) capillary column. Helium was the carrier gas, and the flow‐rate was 0.8 mL/min. The temperature programming started at 100 **°**C for 1 min, ramped up to 160 °C at 10 °C/min, then ramped up to 220 °C at 2 °C/min and finally ramped up to 270 °C at 15 °C/min (maintained at 270 °C for 1 min). The temperature of the injector, the interface and the lines was 250 **°**C. Injections of 0.5 μL were performed in splitless mode. EI mass spectra were recorded using an electron energy of 70 eV, an acceleration voltage of 8 kV and a resolving power of 1000. The trap current was 200 μA, and the magnet scan rate was 1s/decade over a 600–38 *m/z* range. The temperature of ion source was 250 °C. Sugar linkage analysis was deduced from the EI‐MS fragmentation patterns of partially methylated alditol acetate derivatives according to http://www.ccrc.uga.edu/databases/PMAA.

### Determination of degree of methylesterification of pectins

Pectin fractions were saponified by 0.1M sodium hydroxide at 4 °C during 2 h. Solutions were then neutralized by addition of 0.1M HCl and then used for Klavons titration. Five hundred microlitres of samples was diluted to 1 mL with 0.1M potassium phosphate pH 7.5. One millilitre of alcohol oxidase 1U/mL was then added, and the solution was incubated for 15 min at 25 °C. Two millilitres of 0.02M 2.4‐pentanedione in 0.05M acetic acid and 0.2M ammonium acetate was added, and the mixture was heated at 60 °C for 15 min. Absorbance at 412 nm was then recorded and converted into μg/mL of methanol with respect to a calibration curve.

### Determination of degree of acetylesterification of pectins

Pectin acetylation was determined using a Megazyme acetic acid assay kit (E‐ACETRM) after saponification of pectins and monitoring of released acetic acid according to the supplier instructions.

### Xyloglucan fingerprinting

Two milligrams of AIR or hemicellulose fractions was digested with 4U of endo‐beta‐glucanase (Megazyme, Ireland), in sodium acetate buffer 0.01M pH 5 at 37 °C overnight. After addition of three volumes of cold 100% ethanol, the soluble fraction containing xyloglucan fragments was collected by centrifugation at 5.000 ***g***. Supernatant was dried and then dissolved in 100 μL of 0.1% TFA. One microlitre was spotted on a MALDI‐TOF plate with DHB as matrix at 5 mg/mL in ACN ‐ TFA 0.1% (70:30, v:v). Spectra were recorded on a Voyager DE‐Pro from AB Sciex in positive reflector mode and accumulation of 3000 laser shots. MALDI‐TOF MS/MS experiments were performed using a Bruker Autoflex III mass spectrometer equipped with a frequency‐tripled Nd:YAG laser (355 nm), the Flex control 3.3 and Flex Analysis 3.3 software package (Bruker Daltonics, Bremen, Germany). For acquisitions in the tandem time‐of‐flight mode, the precursor ions were accelerated to a kinetic energy of 8 kV and selected in a timed ion gate. The fragment ions generated by laser‐induced decay (LID) of the precursor were accelerated to a kinetic energy of 19 kV in the LIFT cell was analysed after the ion reflector passage.

### Xylan fingerprinting

Search for xylan fragments was performed according to the protocol carried out for xyloglucan fingerprinting using ten microlitres of Xylanase M6 (Megazyme, Ireland) as endoglycanase.

### AGP quantification by rocket gel electrophoresis

Soluble fractions resuspended at 9 mg/mL were spotted on a 1% agarose gel containing Yariv reagent at 50 μg/mL. Gel was then placed under 200V electric field overnight. Quantification was carried out using a calibration curve obtained with arabic gum displaced inside the gel. The area of trapped AGPs was calculated by ImageJ software.

### Determination of lignin deposition

Lignin deposition was monitored by staining *N. benthamiana* leaves with phloroglucinol. Leaves were washed with 100% ethanol during three days to remove pigments. Then, leaves were incubated with 2.5% phloroglucinol in 70% ethanol overnight at room temperature. Revelation of the phloroglucinol staining was then performed by incubation in 37% hydrochloric acid during 5 min.

### Quantification of phenolic compounds

In the xyloglucan‐enriched fractions obtained after endoglucanase digestion precipitated with absolute ethanol, a silylation was performed through a treatment with BSTFA/TMCS (Supelco, Sigma Aldrich) in dry pyridine solvent for 30 min at 80 °C. Samples were then dried and resuspended in cyclohexane before being analysed by GC‐MS Hewlett‐Packard 6890 series gas chromatograph coupled to the Autospec mass spectrometer (Micromass, Manchester, UK) equipped with the Opus 3.1 data system. Separations were performed using a Zebron Z5‐MSi (30 m, 0.25 mm id, 0.25 μm film thickness, Phenomenex) capillary column. Helium was the carrier gas, and the flow rate was 0.8 mL/min. The temperature programming started at 100 **°**C for 1 min, ramped up to 160 °C at 10 °C/min, then ramped up to 220 °C at 2 °C/min and finally ramped up to 270 °C at 15 °C/min (maintained at 270 °C for 1 min). The temperature of the injector, the interface and the lines was 250 **°**C. Injections of 0.5 μL were performed in splitless mode. EI mass spectra were recorded using an electron energy of 70 eV, an acceleration voltage of 8 kV and a resolving power of 1000. The trap current was 200 μA, and the magnet scan rate was 1s/decade over the 600–38 *m/z* range. The temperature of ion source was 250 °C. Identification of phenolic compound was based on the injection of standards of each of them. Quantification was then performed thanks to a ratio between area under curve of the peaks of interest and peak of xylose.

### Statistical analyses

Statistical analyses were performed using the R software. The data were first normalized according to the experimental internal standard method, and then, statistical tests were run on the basis of three biological replicates and three technical replicates. To study the evolution of the cell wall between the four different conditions and the three different days, an ANOVA was performed. Let Y_cds_ be the analysed signal for the component *s*, condition *c* and the day *d*. Therefore, the model is: Y_cds_ = μ + α_c_ + β_d_ + γ_cd_ + ε_cds_, where α_c_ is the condition factor, β_d_ is the day factor, γ_cd_ is the condition and day interaction, and ε_cds_ is the Gaussian residues. The effect between the different plant groups was considered significant when a *P*‐value lower than 0.05 was obtained. When *P*‐value was under 0.05, Bonferroni post‐tests were further performed to specify whether the difference was coming from the condition factor, the day factor or from their synergy.

## Supporting information


**Figure S1** Western blot analysis of H1‐VLPs produced in plants at day 7. Lanes 1, 2 and 3 of the gel were loaded with 100 μg of proteins isolated from three biological replicates.

**Figure S2** MALDI‐TOF MS/MS of the precursor ion *m/z* 1331.7 assigned to sodium adduct of XSGGG and its pattern of fragmentation.

**Table S1** Monosaccharide compositions of AIR extracted from leaves collected at day 1, 4 and 7. nd: not detected.
**Table S2A** Relative proportion of xyloglucan structures found by MALDI‐TOF MS analysis of hemicellulose fraction treated by endoglucanase. nd: not detected, H: hexose, P: pentose.
**Table S2B** Structure of xyloglucan fragments detected after endoglucanase treatment performed on AIR. H: hexose, P: pentose; A: Acetyl group; ?: Unknown structure; *: Structure confirmed by MS‐MS analysis. Nomenclature for proposed structures: G: nonsubstituted Glc unit, G: acetylated Glc, X: Glc substituted by α‐d‐Xyl*p*‐(1→6) residue, S: Glc substituted by α‐L‐Ara*f*‐(1→2)‐α‐D‐Xylp‐(1→6) side chain and T: Glc substituted by α‐L‐Ara*f*‐(1→3)‐α‐L‐Ara*f*‐(1→2)‐α‐D‐Xylp‐(1→6) side chain.
**Table S2C** Relative proportion of xyloglucan fragments detected after endoglucanase treatment performed on AIR. nd: not detected.
