# Supplementary material for: Cell wall biochemical alterations during Agrobacterium‐mediated expression of haemagglutinin‐based influenza virus‐like vaccine particles in tobacco
Source: Plant Biotechnol J. 2017 Jan 5;15(3):285–96. doi: 10.1111/pbi.12607 (PMC5316917; doi:10.1111/pbi.12607)
Supplement: Supplementary file 1 — Figure S1 Western blot analysis of H1‐VLPs produced in plants at day 7. Lanes 1, 2 and 3 of the gel were loaded with 100 μg of proteins isolated from three biological replicates. [file PBI-15-285-s002.docx]

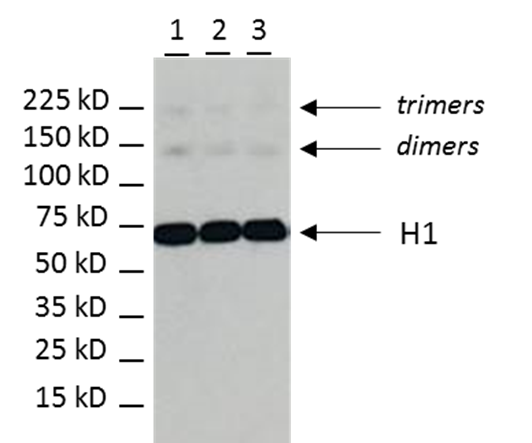


Supplemental Figure 1. Western blot analysis of H1-VLPs produced in plants at day 7. Lanes 1, 2 and 3 of the gel were loaded with 100 µg of proteins isolated from three biological replicates.
