# Supplementary material for: Cell wall biochemical alterations during Agrobacterium‐mediated expression of haemagglutinin‐based influenza virus‐like vaccine particles in tobacco
Source: Plant Biotechnol J. 2017 Jan 5;15(3):285–96. doi: 10.1111/pbi.12607 (PMC5316917; doi:10.1111/pbi.12607)
Supplement: Supplementary file 2 — Figure S2 MALDI‐TOF MS/MS of the precursor ion m/z 1331.7 assigned to sodium adduct of XSGGG and its pattern of fragmentation. [file PBI-15-285-s003.docx]

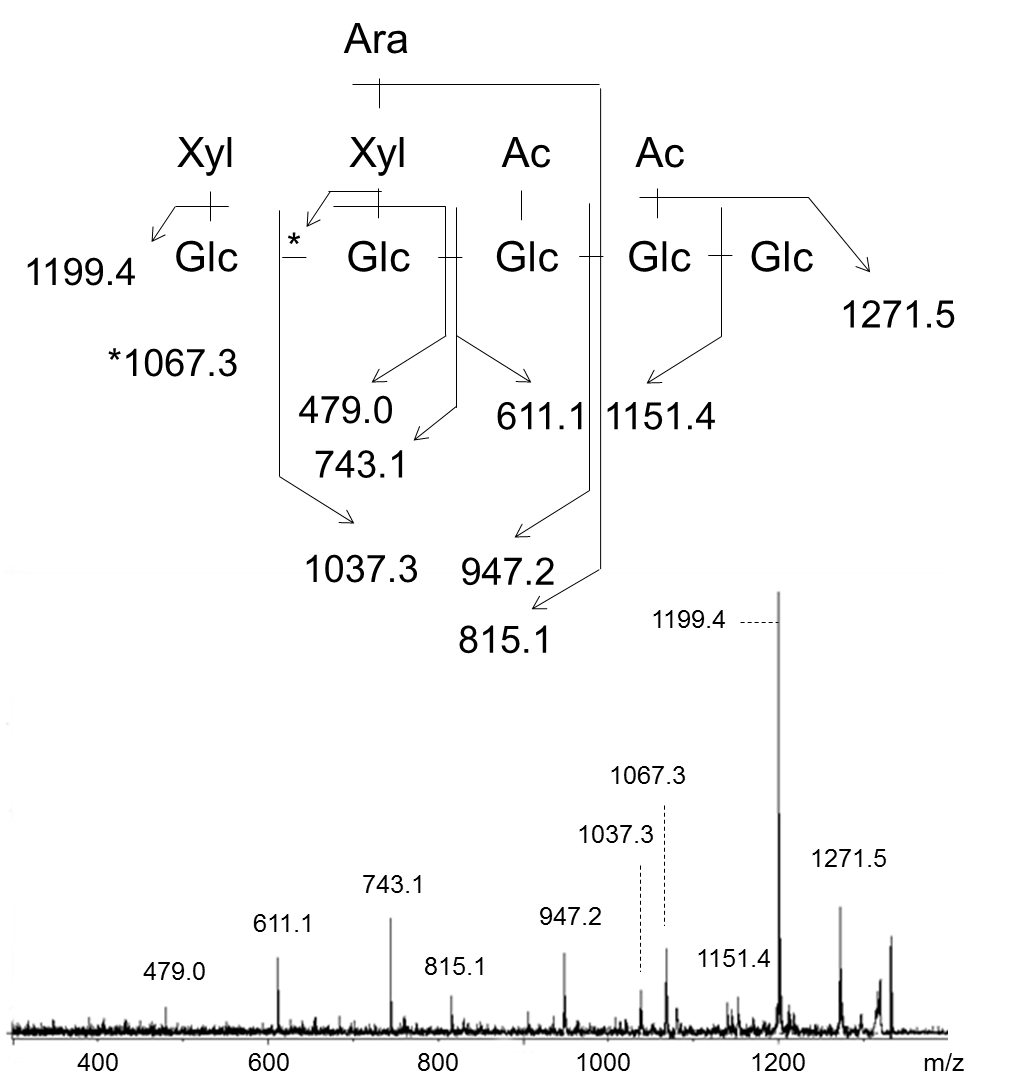
Supplemental figure 2. MALDI-TOF MS/MS of the precursor ion *m/z* 1331.7 assigned to sodium adduct of XSGGG and its pattern of fragmentation.
