# Supplementary material for: Cell wall biochemical alterations during Agrobacterium‐mediated expression of haemagglutinin‐based influenza virus‐like vaccine particles in tobacco
Source: Plant Biotechnol J. 2017 Jan 5;15(3):285–96. doi: 10.1111/pbi.12607 (PMC5316917; doi:10.1111/pbi.12607)
Supplement: Supplementary file 3 — Table S1 Monosaccharide compositions of AIR extracted from leaves collected at day 1, 4 and 7. nd: not detected. Table S2A Relative proportion of xyloglucan structures found by MALDI‐TOF MS analysis of hemicellulose fraction treated by endoglucanase. nd: not detected, H: hexose, P: pentose. Table S2B Structure of xyloglucan fragments detected after endoglucanase treatment performed on AIR. H: hexose, P: pentose; A: Acetyl group; ?: Unknown structure; *: Structure confirmed by MS‐MS analysis. Nomenclature for proposed structures: G: nonsubstituted Glc unit, G: acetylated Glc, X: Glc substituted by α‐d‐Xylp‐(1→6) residue, S: Glc substituted by α‐L‐Araf‐(1→2)‐α‐D‐Xylp‐(1→6) side chain and T: Glc substituted by α‐L‐Araf‐(1→3)‐α‐L‐Araf‐(1→2)‐α‐D‐Xylp‐(1→6) side chain. Table S2C Relative proportion of xyloglucan fragments detected after endoglucanase treatment performed on AIR. nd: not detected. [file PBI-15-285-s001.docx]

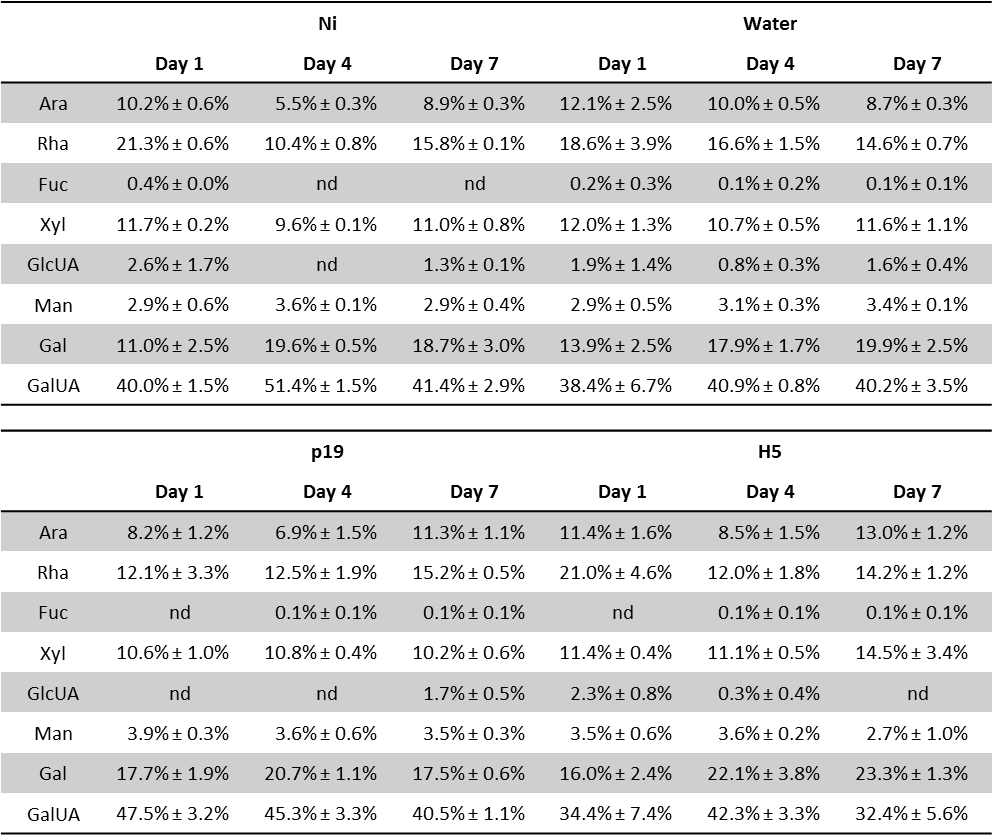


Supplemental table 1. Monosaccharide compositions of AIR extracted from leaves collected at day 1, 4 and 7. nd: not detected.


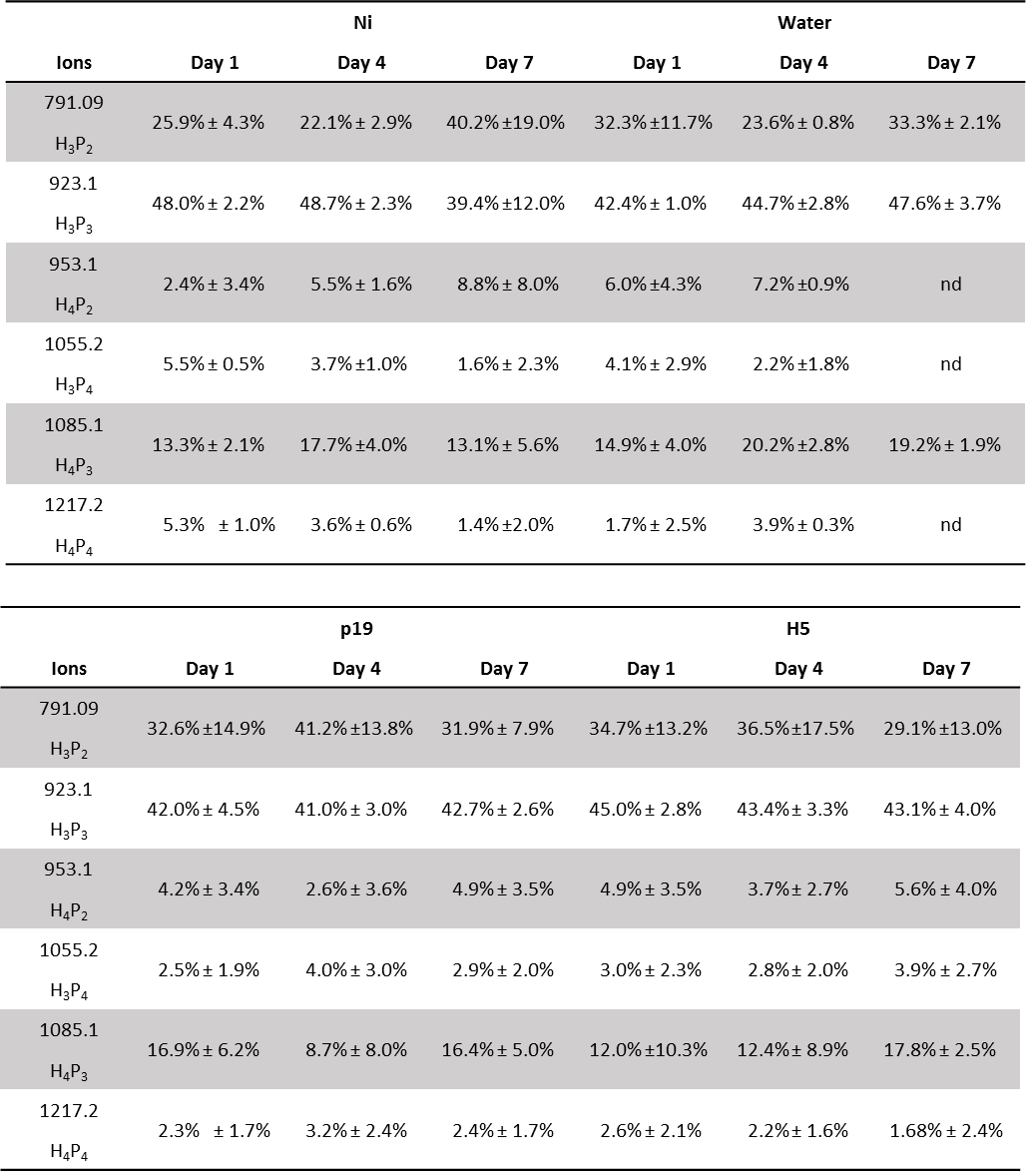
Supplemental table 2A. Relative proportion of xyloglucan structures found by MALDI-TOF MS analysis of hemicellulose fraction treated by endoglucanase. nd: not detected, H: hexose, P: pentose.


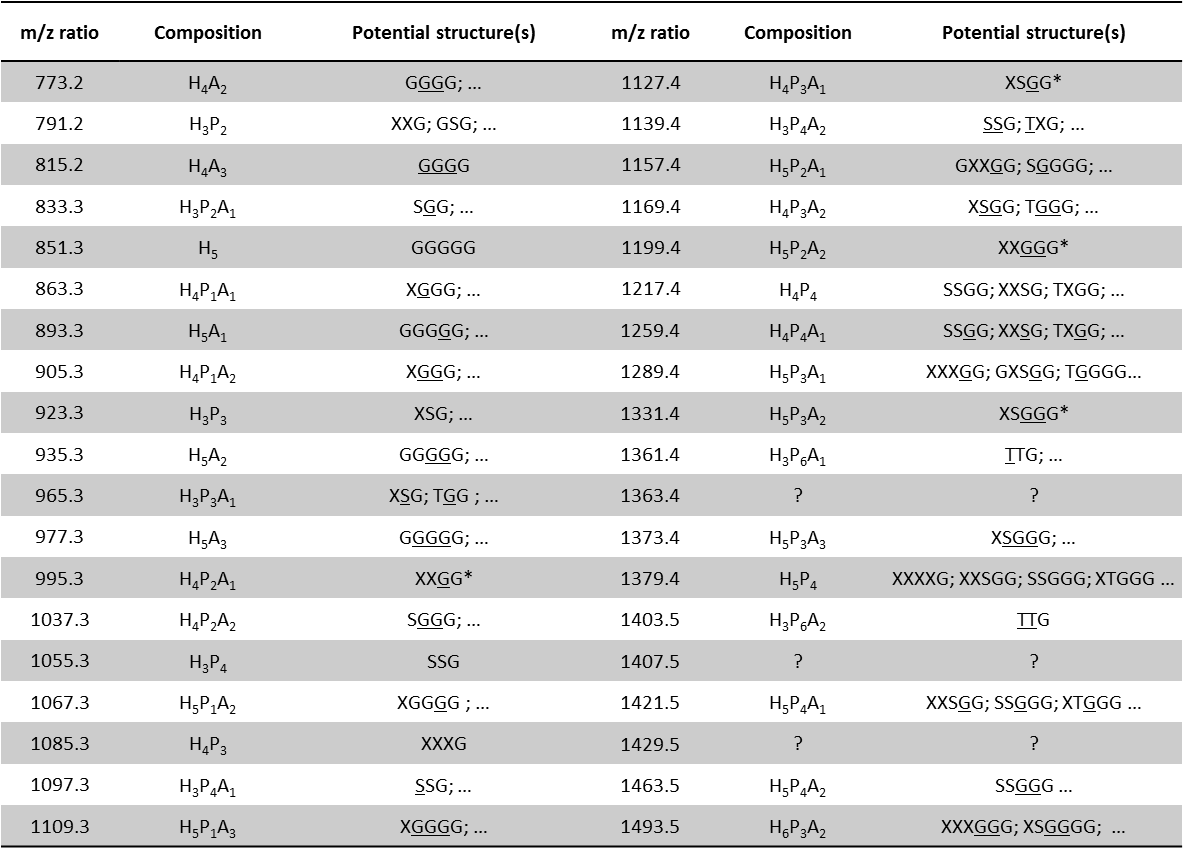
Supplemental table 2B. Structure of xyloglucan fragments detected after endoglucanase treatment performed on AIR. H: hexose, P: pentose; A: Acetyl group; ?: Unknown structure; *: Structure confirmed by MS-MS analysis. Nomenclature for proposed structures: G: non substituted Glc unit, G: acetylated Glc, X: Glc substituted by α-D-Xyl*p*-(1→6) residue, S: Glc substituted by α-L-Ara*f*-(1→2)-α-D-Xylp-(1→6) side chain and T: Glc substituted by α-L-Ara*f*-(1→3)-α-L-Ara*f*-(1→2)-α-D-Xylp-(1→6) side chain.


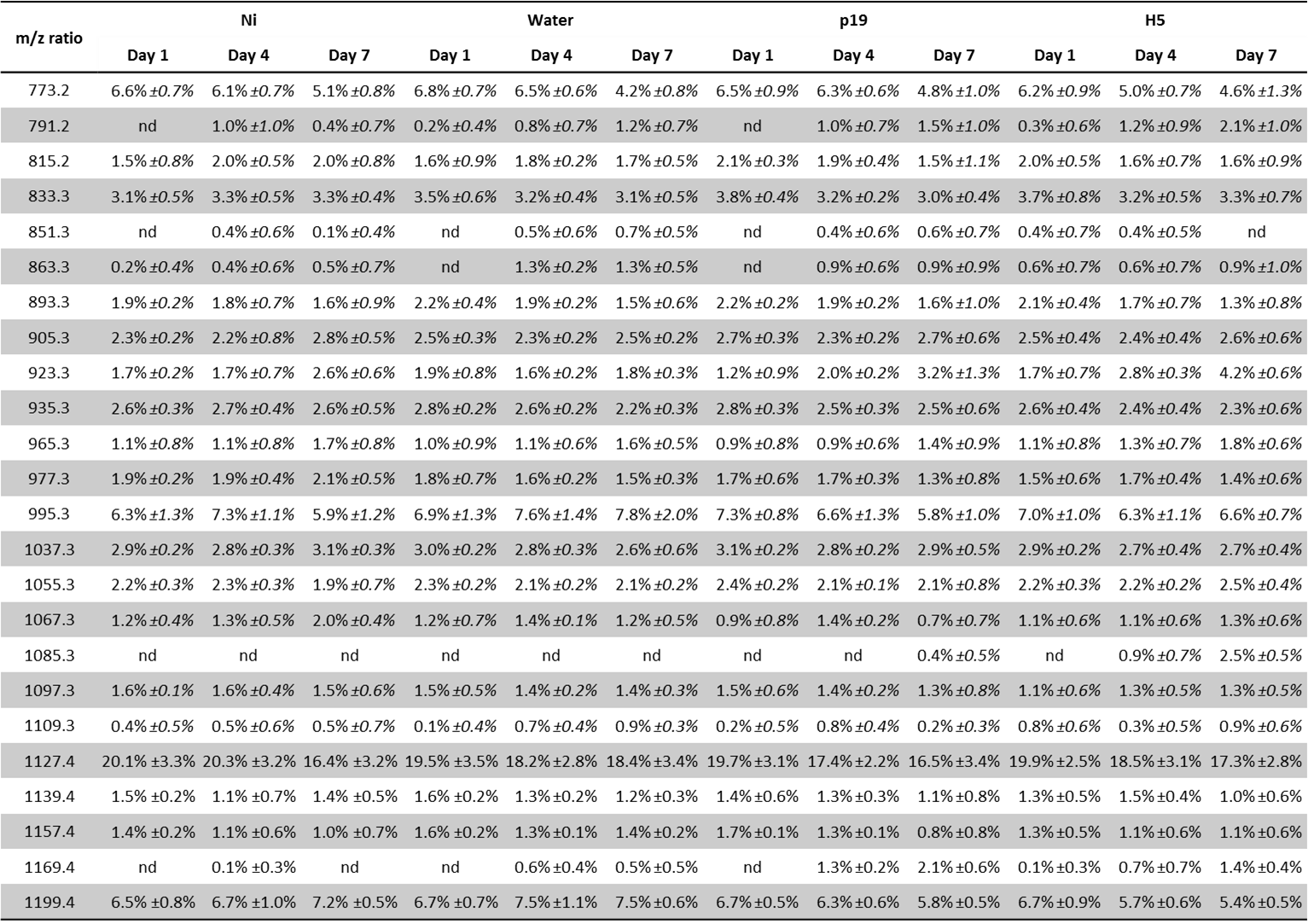


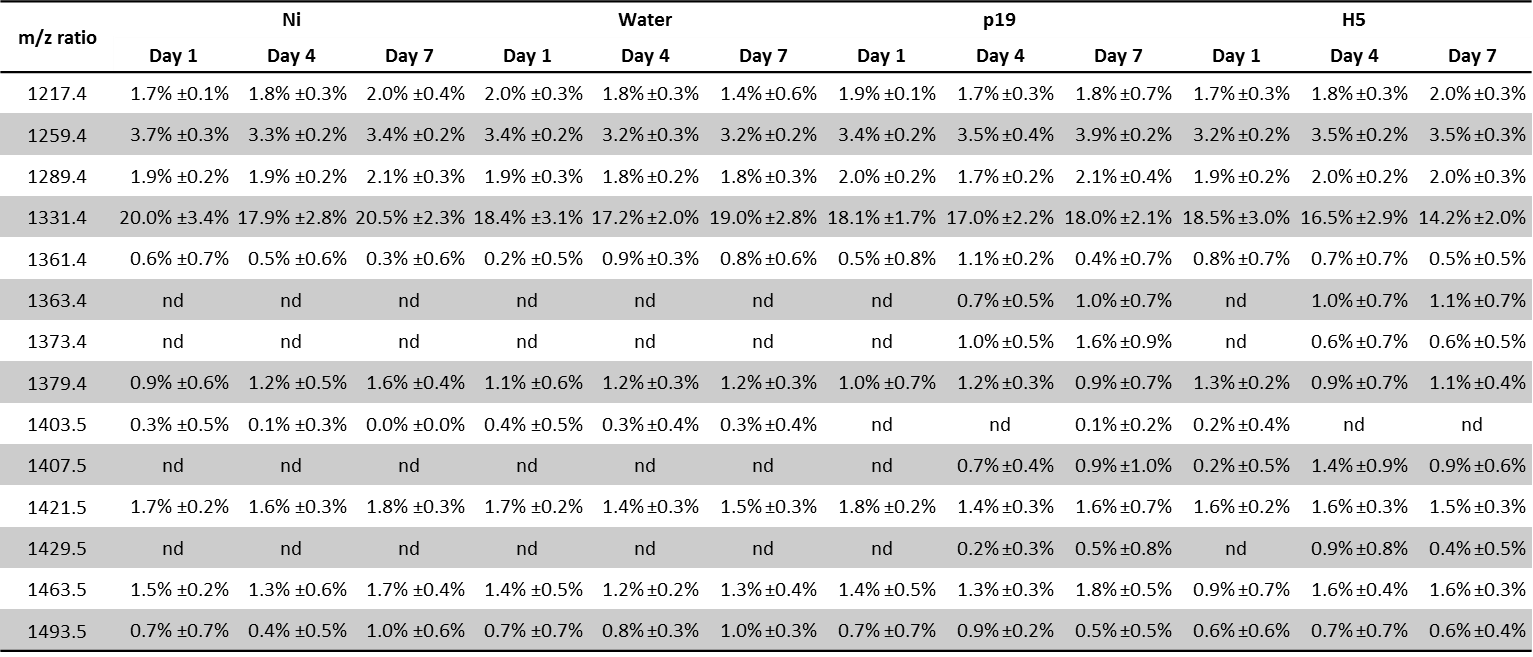
Supplemental table 2C. Relative proportion of xyloglucan fragments detected after endoglucanase treatment performed on AIR. nd: not detected.
